# Bayesian Network Analysis Reveals Alterations to Default Mode Network Connectivity in Individuals at Risk for Alzheimer's Disease

**DOI:** 10.1371/journal.pone.0082104

**Published:** 2013-12-06

**Authors:** Rui Li, Jing Yu, Shouzi Zhang, Feng Bao, Pengyun Wang, Xin Huang, Juan Li

**Affiliations:** 1 Center on Aging Psychology, Key Laboratory of Mental Health, Institute of Psychology, Chinese Academy of Sciences, Beijing, China; 2 Magnetic Resonance Imaging Research Center, Institute of Psychology, Chinese Academy of Sciences, Beijing, China; 3 School of Psychology, Southwest University, Chongqing, China; 4 Beijing Geriatric Hospital, Beijing, China; 5 Beijing Anding Hospital, Capital Medical University, Beijing, China; Beijing Normal University, China

## Abstract

Alzheimer's disease (AD) is associated with abnormal functioning of the default mode network (DMN). Functional connectivity (FC) changes to the DMN have been found in patients with amnestic mild cognitive impairment (aMCI), which is the prodromal stage of AD. However, whether or not aMCI also alters the effective connectivity (EC) of the DMN remains unknown. We employed a combined group independent component analysis (ICA) and Bayesian network (BN) learning approach to resting-state functional MRI (fMRI) data from 17 aMCI patients and 17 controls, in order to establish the EC pattern of DMN, and to evaluate changes occurring in aMCI. BN analysis demonstrated heterogeneous regional convergence degree across DMN regions, which were organized into two closely interacting subsystems. Compared to controls, the aMCI group showed altered directed connectivity weights between DMN regions in the fronto-parietal, temporo-frontal, and temporo-parietal pathways. The aMCI group also exhibited altered regional convergence degree in the right inferior parietal lobule. Moreover, we found EC changes in DMN regions in aMCI were correlated with regional FC levels, and the connectivity metrics were associated with patients' cognitive performance. This study provides novel sights into our understanding of the functional architecture of the DMN and adds to a growing body of work demonstrating the importance of the DMN as a mechanism of aMCI.

## Introduction

Alzheimer's disease (AD) is a neurodegenerative disorder characterized by cognitive decline and progressive dementia [1], and is thought to be caused by aberrant connections between cerebral regions involved in cognitive functioning [2], [3]. Amnestic mild cognitive impairment (aMCI) is the transitional stage between normal aging and early AD, and individuals diagnosed with aMCI are at high risk for progression to AD [4]. Numerous neuroimaging studies have demonstrated that patients with aMCI also have alterations to regional [5] and functional [6]–[8] neural networks, as well as to whole-brain connectivity [9].

Functional magnetic resonance imaging (fMRI) of the neural activity occurring in the default mode network (DMN) under resting state has recently attracted attention as a novel means to understand the mechanism of AD [10], [11], and as a potential biomarker to diagnose incipient AD [12]. The DMN comprises a constellation of regions that are more active under resting state than during goal-oriented or attention-demanding tasks; these regions include the medial prefrontal cortex (MPFC), posterior cingulate cortex (PCC), and inferior parietal lobule (IPL) as core regions, and the lateral temporal cortex (LTC) and medial temporal lobe (MTL) as additional regions [10], [13]. Intriguingly, the DMN regions are the typical predilection targets of AD [14], a finding confirmed by the functional [11], [15], [16] and structural [17] connectivity alterations revealed by neuroimaging studies of AD. With respect to the DMN in aMCI, Sorg et al. [18] found that connectivity between bilateral hippocampus and left PCC was selectively decreased in aMCI, while Bai et al. [19], Qi et al. [6] and Jin et al. [20] reported the coexistence of both impairment and compensation within the DMN in aMCI. These studies indicate a potential significance of the DMN as a useful tool for understanding functional abnormalities present in the pathology of aMCI.

Considering the methodology used, most previous fMRI connectivity studies of the DMN in aMCI are, in essence, evaluations of temporal correlations between brain regions within the network, which is generally referred to as functional connectivity (FC) analysis [21]. This is performed using region of interest (ROI) based linear correlation analysis [22], or data-driven independent component analysis (ICA) [6], [18], [20]. Recently, effective connectivity (EC) analyses, which provide insights into directed relationships between brain regions [23], for example, Granger causality analysis (GCA) [24] and Bayesian network (BN) [25], has been employed in resting state fMRI connectivity studies to reveal further characteristics of spontaneous fluctuations [26]–[28]. These methods have also been used in AD studies to evaluate directed connectivity changes [16], [29]. However, whether aMCI patients also exhibit an aberrant EC relationship between DMN regions remains largely unknown.

Here, we employed a combined Group ICA and BN learning approach to explore resting state fMRI effective connectivity changes in the DMN, in patients diagnosed with aMCI. Briefly, Group ICA is the regular method used for FC analysis of resting state brain networks [6], [11], [30]. BN is a directed acyclic graph (DAG) model of joint multivariate probability distributions, that captures the conditional independence relationships among multiple interacting quantities (in this case, brain regions of interest) [31]. BN is used to describe global directed connectivity patterns between brain regions in a data-driven manner, without any prior knowledge of the relationships among them [25]. In the current work, Group ICA was first used for the separation of FC map of DMN, which was then used for the determination of ROIs for EC analysis. Gaussian BN was finally employed to model the EC patterns of these DMN regions. With the use of Group ICA and BN analysis, we aimed to determine the functional organization of DMN regions from the perspective of directed connectivity, and to evaluate whether aMCI alters the EC of DMN, and if so, whether the EC changes were correlated with regional FC levels, and associated with individual clinical and cognitive performance.

## Materials and Methods

### Subjects

Seventeen aMCI patients (7 men and 10 women), and 17 age-, gender-, and education-matched healthy normal control subjects (NC: 9 men and 8 women) participated in this study. All subjects were recruited from the local communities of Beijing, China. Each participant underwent a series of standardized clinical and neuropsychological evaluations, including the Clinical Dementia Rating (CDR) Scale [32], the Mini-Mental State Examination (MMSE) [33], and the Montreal Cognitive Assessment (MoCA) [34]. Participants' cognitive skills in several domains, including visuospatial executive function, attention, delayed recall, and orientation to time and place were assessed using MoCA. All aMCI subjects were diagnosed by experienced neurologists. None of the aMCI patients was taking any specific medications for their condition during the period of the study. Subjects with any history of psychiatric disease, neurological disorder, drug abuse, moderate to serious hypertension, or known systematic disease were excluded. Patients met the diagnostic criteria for aMCI, which include reported and neuropsychologically assessed memory impairments, largely intact activities of daily life, and an absence of dementia [4], [35]. Table 1 shows the detailed demographic data and clinical/cognitive characteristics of these participants.

**Table 1:** Demographics and Clinical/Cognitive Characteristics of the Participants.

Characteristic	NC	aMCI	P value
N (M/F)	17 (7/10)	17 (9/8)	0.49^a^
Age, years	67.9±5.6	70.5±4.5	0.15^b^
Education, years	11.8±3.2	9.8±4.6	0.16^b^
MMSE	28.4±1.4	24.5±3.9	<0.001^b^
CDR	0	0.5	—
MoCA	26.1±1.5	19.2±4.4	<0.001^b^
Visuospatial executive function	4.6±0.7	2.5±1.0	<0.001^b^
Attention	5.9±0.3	4.4±1.5	0.002^b^
Delayed recall	3.2±1.0	1.6±1.6	0.009^b^
Orientation	5.9±0.2	5.3±1.0	0.023^b^

a The *p* value was obtained using a two-tail Pearson chi-square test.

b The *p* value was obtained using a two-sample two-tail *t* test.

### Ethics Statement

This study was approved by the Institutional Review Board of the Institute of Psychology, Chinese Academy of Sciences. All participants provided written, informed consent before taking part in our experiments.

### Data Acquisition

A 3-Tesla Siemens Trio scanner (Erlangen, Germany), equipped for echo planar imaging (EPI), at the Beijing MRI Center for Brain Research was used for image acquisition. During the scan, participants were instructed to lie quietly, keep their eyes closed, and not to think of anything in particular. For each participant, 200 resting state functional volumes were collected, using the following parameters: time repetition (TR) = 2000 ms, time echo (TE) = 30 ms, flip angle  = 90°, field of view (FOV) = 200×200 mm^2^, 33 axial slices, thickness  = 3.0 mm, gap  = 0.6 mm, acquisition matrix  = 64×64, and in-plane resolution  = 3.125×3.125. High-resolution, three-dimensional T1-weighted structural images were acquired for each subject, with the following parameters: 176 slices, acquisition matrix  = 256×256, voxel size  = 1×1×1 mm^3^, TR  = 1900 ms, TE  = 2.2 ms, and flip angle  = 9°.

### Data Pre-processing

Data pre-processing was performed using the Statistical Parametric Mapping program (SPM8, http://www.fil.ion.ucl.ac.uk/spm) and the Data Processing Assistant for Resting State fMRI (DPARSF) V2.0 Basic Edition (http://www.restfmri.net). The first 10 volumes were discarded to allow for equilibration of the magnetic field. The remaining 190 volumes were corrected for the intra-volume acquisition time differences between slices using the Sinc interpolation and were corrected for the inter-volume geometrical displacement due to head motion using a six-parameter (rigid body) spatial transformation. Participants included in this study are with head motions less than 2.0-mm in any direction and 2.0° of any angular motion during the resting-state scan. The aMCI group and NC group were matched on mean head motion (*p*>0.08 in any direction). The functional images were then normalized into the standard space of Montreal Neurological Institute (MNI) using an optimum 12-parameter affine transformation and nonlinear deformations. The normalized volumes were resampled to a voxel size of 3×3×3 mm^3^. Finally the images were spatially smoothed with a 4-mm full width at half maximum (FWHM) Gaussian kernel.

### Group Independent Component Analysis

Group ICA is a data-driven technique which is widely used to separate patterns of task-activated neural networks, image artifacts and physiologically generated independent components (ICs), including resting state networks [30], [36]–[38]. Here, the pre-processed fMRI data from all subjects were entered into the software of Group ICA in the fMRI Toolbox (GIFT, http://icatb.sourceforge.net), for the separation of DMN, and the determination of DMN regions for BN analysis. As there is no consensus on the selection of an optimal number of ICs, we chose to have the analysis output 20 components [11], [30], [39]. The Group ICA program included two rounds of principal component analysis (PCA) for reduction of fMRI data dimension, ICA separation, and back-reconstruction of the ICs and the corresponding mean time course for each subject. The component that covers the main regions of the DMN previously reported [10], [13] was selected for statistical analysis. To derive the Group DMN pattern, we converted the intensity values within each subject's DMN component to *z*-scores, and applied a one-sample *t*-test (*p*<0.05, corrected by false discovery rate [FDR]) to these individual components. To determine the regions for the subsequent EC analysis, we identified key DMN regions as ROIs, each of which was a sphere centered on the local maximum FC cluster, with a radius of 6 mm.

### Gaussian Bayesian Network Analysis

The ROIs identified in the previous process were entered into the BN analysis for the construction of EC patterns of DMN. The averaged resting state fMRI time series over the voxels in each ROI was extracted to represent the time course of the region. In particular, before the extraction of ROIs' time series, detrending and temporal band-pass filtering of the fMRI data was performed in order to reduce the effects of low-frequency drifts and physiological high-frequency noise [40], [41]. As the head movement and several potential nuisance signals were reported to influence the resting-state connectivity [42]–[44], we regressed out the possible sources of artifacts of the fMRI data including the six head-motion profiles, global signal, white matter signal, and cerebrospinal fluid (CSF) signal. The residual volumes were retained for the inter-ROI effective connectivity analysis.

A BN is a graphical representation of a joint probability distribution over a set of random variables. It is a directed acyclic graph (DAG), with nodes corresponding to the research variables, and arcs denoting conditional dependence relationships. Each node is quantified by its conditional probability, given its parent nodes in the network. The absence of arcs between nodes in a BN refers to conditional independencies among them. The BN graph encodes the Markov assumption; that is, each node is independent of its non-descendants, given its parents in the network [31]. Here, nodes in the BN represent ROIs from DMN, the time series from which is assumed to follow a linear Gaussian conditional distribution. Consider a set 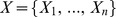 of random variables where variable 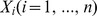 represents the i*th* ROI, the conditional probability density of 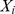 given its parents 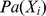 can be given by
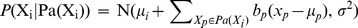
(1)in which 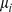 is the unconditional mean of 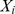, 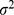 is the conditional variance of 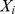 given its parents, and 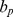 is a linear connectivity coefficient from the parent node 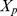 to 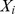 that quantifies the strength of the relationship between them [45]. The joint probability distribution of 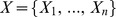, namely,
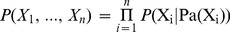
(2)is thus a multivariate Gaussian. A determined linear Gaussian BN is in fact equivalent to a set of multivariate linear regression equations. Each node 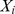 in a Gaussian BN can be viewed as the linear regression of its parents 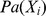 [46].

The BN method requires no assumption of any prior knowledge of the relationship between research variables, and can provide a global connectivity characterization of a system automatically learned from data in an exploratory manner. We employed the Bayesian information criterion (BIC) [47] based search-and-score approach to learn the EC among DMN regions. The BN model that optimizes the BIC score among the space of possible candidates was identified as the best fit network. The L1-Regularization Paths algorithm [48], and the maximum likelihood (ML) estimate implemented in the collections of Matlab functions written by Murphy et al. (http://www.cs.ubc.ca/~murphyk/Software), were used for learning the DAG structure and parameters of the BN model, respectively, for the NC and aMCI groups.

For the constructed BN pattern of DMN regions, it was also of interest to examine the network features on the basis of the determination of directed connections among DMN regions. The BN is particularly useful for characterizing processes composed of locally interacting nodes [31]. Within the framework of BN, the activity of each DMN region is directly dependent on the activity of its several parent regions (ingoing connections), and regions with more parents exhibit a higher regional dependence degree in the network. Therefore, we defined a network measure, the convergence index (CI) quantified as the number of parents (i.e., the number of ingoing connections), for each region in order to describe the regional dependence degree. Regions with a higher CI have more parents, depend more on the activity of the network, and might be more pivotal within the DMN. We further differentiated these DMN regions into those having a higher CI and those having a lower CI, according to whether or not their dependence degree was above or below the average degree value of all the regions.

### Statistical Analysis

Before examining EC changes in aMCI, we first conducted ROI analyses to examine the between-group FC changes, and their correlations with clinical/cognitive variables in each ROI. As the *z*-score reflects the correlation degree of a given voxel's time series to the mean time course of the component, we calculated the average *z*-score of voxels within each ROI for each subject in order to quantify the level of FC in these regions. To determine the between-group FC differences in the DMN, a two-sample *t*-test (*p*<0.05) was performed for each ROI. Correlations between FC in these DMN regions, and cognitive variables including MMSE and MoCA scores were also examined.

EC changes to the DMN in aMCI were examined in two ways. Firstly, to determine the changes of directed connections between DMN regions in aMCI, a randomized permutation test was performed [16], [49]. The differences in connection weight coefficients between NC and aMCI were taken as the statistical measures. We first calculated between-group differences of the connection coefficients, and then obtained the reference distributions of these statistics by randomly rearranging all of the 34 fMRI datasets into two groups, reconstructing the BN models, and recalculating the differences of these connection weights between the two randomized groups (1000 permutations). Finally, the probabilities of BN connections in NC group that were higher than those in aMCI group, as well as probabilities of connections in aMCI group that were stronger than those in NC group (*p*<0.05) were examined, and the type-I errors of between group differences for each connection were reported. Secondly, to determine changes in regional dependence degree in each DMN region in aMCI, we compared the CI of each ROI between NC and aMCI using a two-sample *t*-test (*p*<0.05).

To examine whether the EC changes in aMCI are associated with regional FC levels, we calculated the Pearson correlation (*p*<0.05) between regional dependence degree (CI) and regional FC value for each region that showed an EC difference in the aMCI group.

To examine the relationship between network connectivity metrics and clinical/cognitive measures in aMCI group, we calculated the Pearson correlation (*p*<0.05) between CI values in regions with altered EC, and patients' global performance (MMSE/MoCA); we also calculated the Spearman correlation (*p*<0.05) between CI and individual cognitive performance in the domain of visuospatial executive function, attention, delayed recall, and orientation function.

## Results

### Demographic, Clinical, and Cognitive Characteristics

There were no significant differences in age (*p* = 0.15), gender (*p* = 0.49), or years of education (*p* = 0.16) between the NC and aMCI groups. The aMCI group had significantly lower scores on the MMSE and MoCA tests than NC group (*p*<0.001). The aMCI group also had significantly lower scores for visuospatial executive (*p*<0.001), attention (*p* = 0.002), delayed recall (*p* = 0.009), and orientation (*p* = 0.023) abilities than NC group.

### Functional Connectivity Mapping of the Default Mode Network

**Figure 1:**
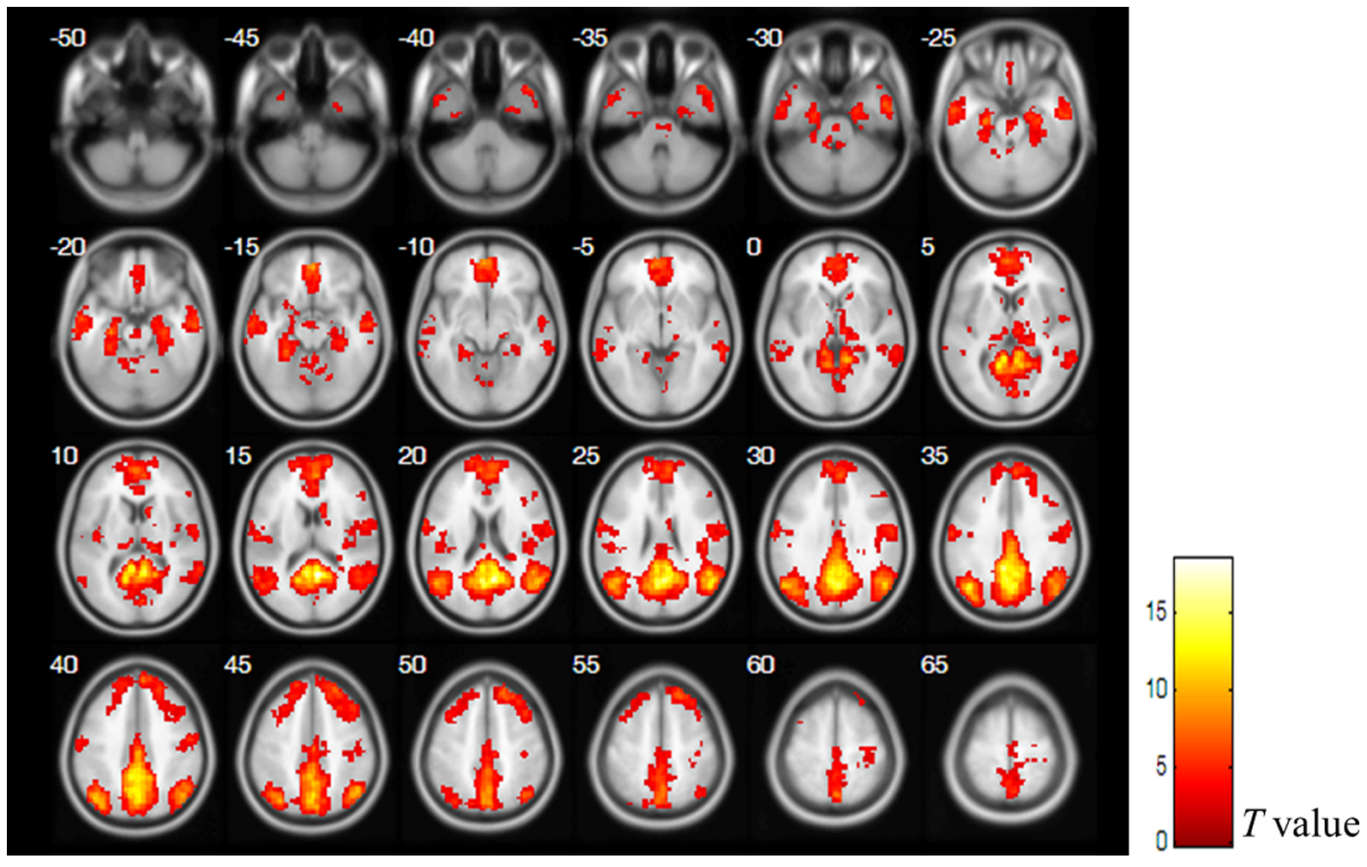
The functional connectivity map of the DMN. The statistical map was derived from a one sample t-test (*p*<0.05, corrected by FDR) of all subjects' DMN components. Bar at the right shows *T*-values.

Figure 1 shows the FC map of DMN that was extracted from Group ICA. To determine the regions for the subsequent EC modeling of the DMN, we choose 9 regions that showed significant FC (*p*<0.05, corrected by FDR) as ROIs. They are PCC, dorsal MPFC (dMPFC), ventral MPFC (vMPFC), left IPL (LIPL), right IPL (rIPL), left LTC (lLTC), right LTC (rLTC), left hippocampus (lHC), and right hippocampus (rHC). The significant DMN cluster locations are shown in Table 2.

**Table 2:** Regions showing significant FC in the DMN.

Region	Peak MNI location			Brodmann's areas	Peak T
PCC	9	−54	15	23, 30, 31	18.6
dMPFC	6	57	18	9, 10, 24, 32	8.7
vMPFC	0	48	−15	10, 24, 32	9.7
lIPL	−45	−69	36	39, 40	13.6
rIPL	54	−60	24	39, 40	13.1
lLTC	−57	−6	−21	20, 21	7.0
rLTC	60	−12	−21	20, 21	6.7
lHC	−27	−21	−21	28, 35, 36	9.2
rHC	27	−21	−21	28, 35, 36	6.7

### Effective Connectivity Mapping of the Default Mode Network

**Figure 2:**
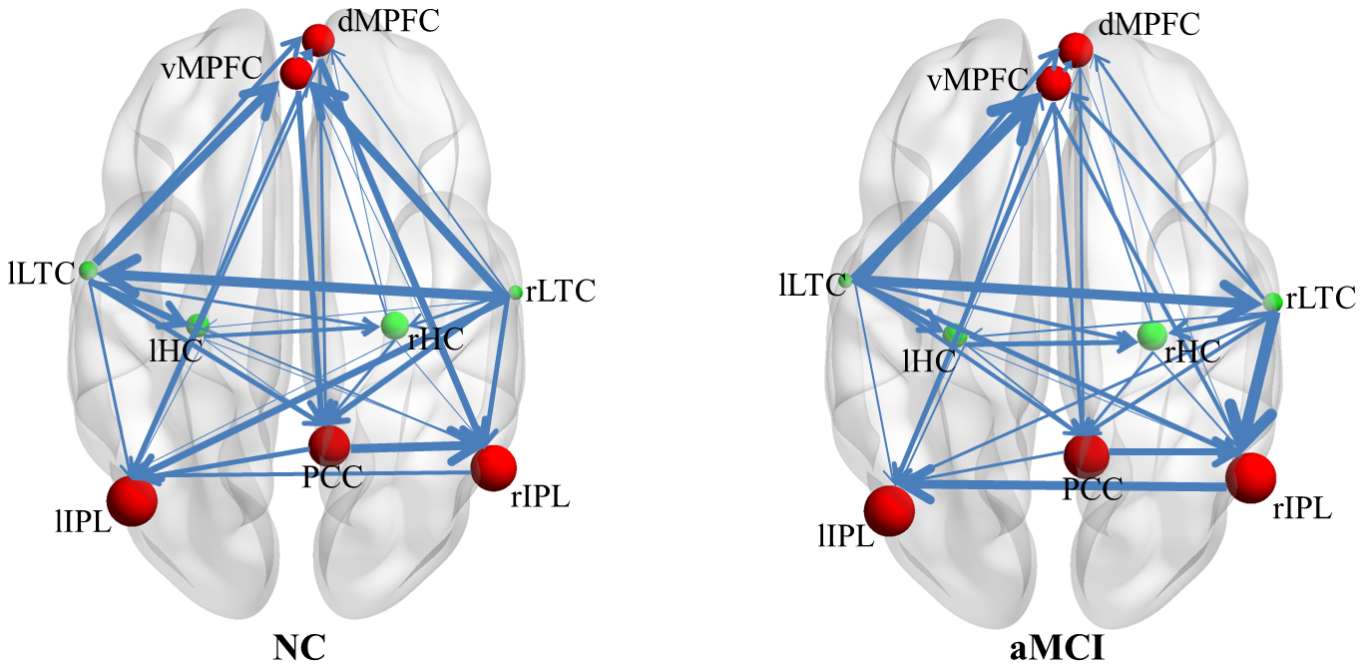
The directed connectivity of the DMN. The DMN regions are graphically represented with connections depicting conditional dependencies. Line width is proportional to the connection weight, and node size is proportional to the regional CI. Red and green nodes indicate the regions with higher and lower CI than the average, respectively.

The Gaussian BN approach was applied to characterize the directed connectivity relationships among the 9 DMN regions. Figure 2 shows the BIC-optimized BN connectivity patterns of the DMN in both the NC and aMCI groups. The DAG demonstrations of the EC patterns were constructed using arcs of varying line width to indicate the direction and strength of the conditional dependence relationships. Intuitively, the group-level EC patterns in the NC and aMCI groups seem very similar, except for one connection (rLTC→lLTC) which changed directionality, and one small-weighted connection (rHC→lIPL) which disappeared in aMCI.

We also calculated the CI at each node, in order to differentiate the regional convergence degree among these ROIs. Figure 2 shows that the PCC, dorsal and ventral MPFC, and bilateral IPL (shown as red nodes) have a higher CI in both groups; each of them has more ingoing connections, not only from themselves but also from other regions. While the bilateral LTC and bilateral HC (shown as green nodes) had a lower CI in both groups, each of them had only ingoing connections from each other, with no ingoing connections from the PCC, MPFC, or IPL connecting to them.

### FC Changes and Their Relationship with MMSE/MoCA in aMCI

**Figure 3:**
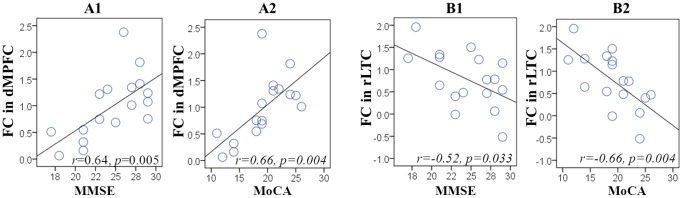
Relationship between FC and individual performance in aMCI. Scatter plots show the significantly positive correlation between FC in the dMPFC with MMSE (*A1*) and MoCA (*A2*) scores, and the significant negative correlation between the rLTC with MMSE (*B1*) and MoCA (*B2*) scores. Each circle represents data from a single participant.

We examined the FC differences in the 9 ROIs between the NC and aMCI groups. The between-group comparison of the FC in these ROIs showed that there was no significant difference in any of the 9 DMN regions (*p*>0.05). However, we found that the FC values in two regions, the dMPFC and the rLTC, were significantly correlated with patients' global clinical and cognitive performance. The FC values in the dMPFC were positively correlated with MMSE (*r* = 0.64, *p* = 0.005) and MoCA (*r* = 0.66, *p* = 0.004) scores, while in the rLTC, the FC values were negatively correlated with MMSE (*r* = −0.52, *p* = 0.033) and MoCA (*r* = −0.66, *p* = 0.004) scores (Figure 3).

### EC Changes in aMCI

The alterations to the EC of the DMN in aMCI were demonstrated in two ways, including changes in connectivity weight between regions and changes in regional convergence degree.

**Figure 4:**
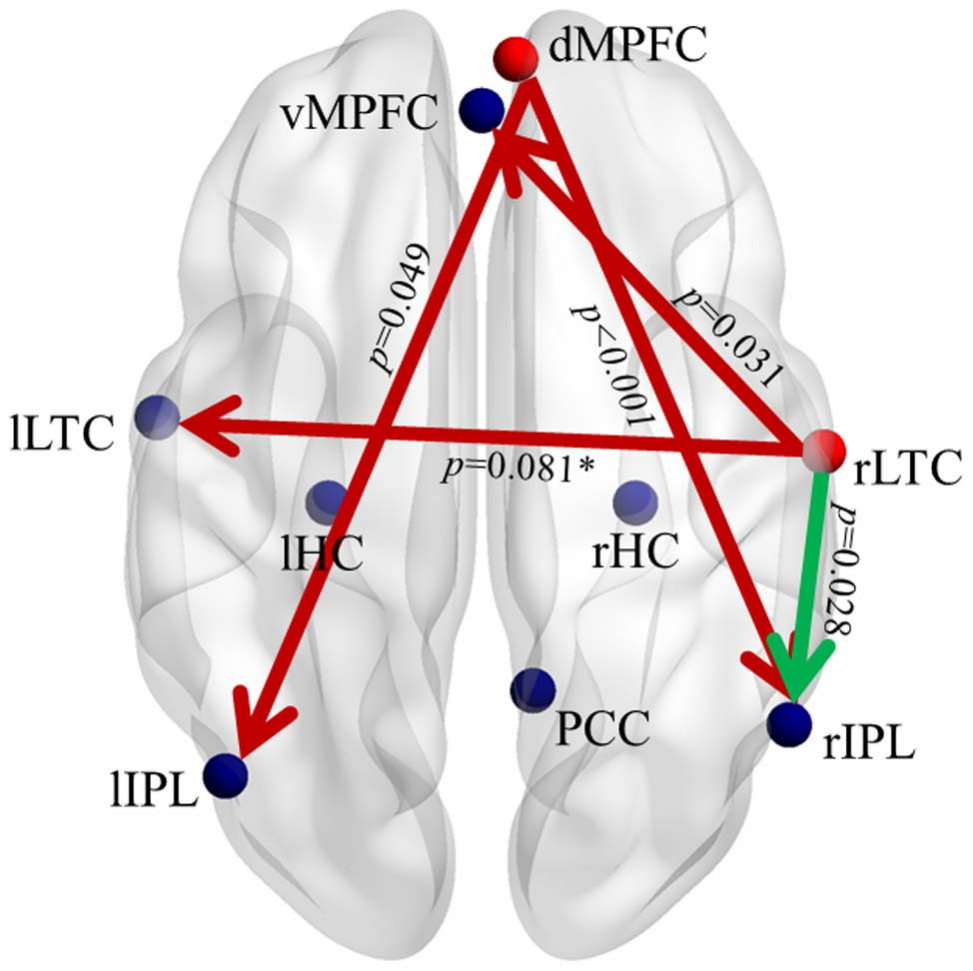
Changes in directed connectivity between DMN regions in aMCI. Red and green connections indicate increased and decreased connectivity weights, respectively, in aMCI compared to controls. All the aberrant connections generate from the dMPFC or the rLTC, which are shown in red nodes. The type-I error of having a between group difference is shown next to each connection.

Figure 4 shows the between-group differences in connectivity weights. Compared to controls, the aMCI group had decreased connectivity weight of the connections dMPFC→lIPL, dMPFC→rIPL, and rLTC→vMPFC, and increased connectivity weight of the rLTC→vMPFC connection (*p*<0.05). Although the connection between the bilateral LTC changes direction, and the rHC→lIPL connection disappears in aMCI, we did not find any significant between-group differences in connectivity weight for these connections at the group level (*p*>0.05). There was only a trend towards decreased rLTC→lLTC connectivity in aMCI (*p* = 0.081).

**Figure 5:**
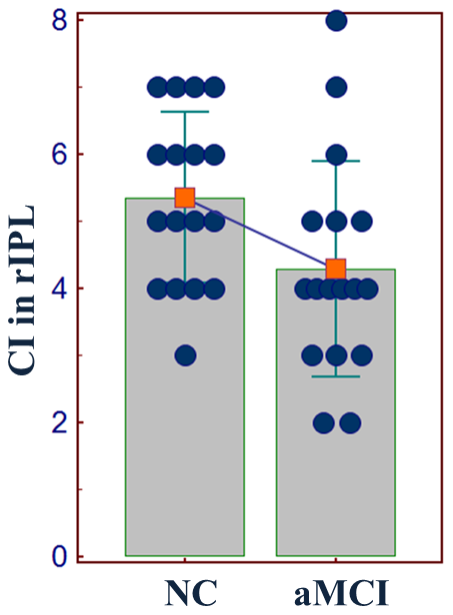
Scatter plot of the CI in the rIPL. The circles represent the data from individual subjects. The histogram and the error bar show the within-group mean value, and the standard deviation. A declining line between the two histograms indicates a significantly decreased CI in the rIPL from NC to aMCI.

Figure 5 shows the changes of regional convergence degree in aMCI. We found that in aMCI group, the local convergence degree in the rIPL was altered. As shown in Figure 5, the CI in rIPL is significantly decreased from 5.4±1.3 in NC to 4.3±1.6 in aMCI (*p* = 0.041). There was no significant change in CI in other regions in aMCI (*p*>0.05).

### Relationship between EC and FC in aMCI

**Figure 6:**
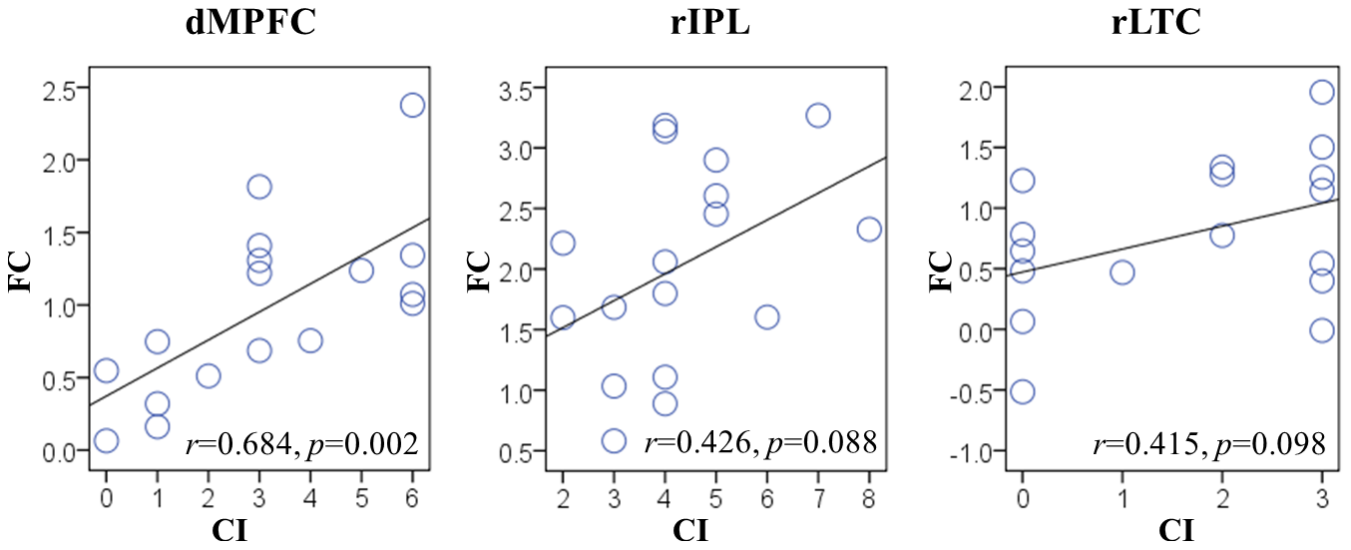
Correlation analysis between two fMRI connectivity attributes in aMCI. Scatter plots show the significant positive correlation between FC and CI in the dMPFC. (*p*<0.05), and trending positive correlations between FC and CI in the rIPL and rLTC. (0.05≤*p*≤0.10). Each circle represents data from a single subject.

For the 5 regions with significantly altered EC in aMCI, including the dorsal and ventral MPFC, bilateral IPL, and rLTC, we examined the correlation between the local convergence degree and regional FC level, in order to examine the intrinsic relationship between two different connectivity attributes in aMCI. We found a significantly positive correlation between CI values and FC levels in the dMPFC (*r* = 0.684, *p* = 0.002), and trending positive correlations in the rIPL (*r* = 0.426, *p* = 0.088) and rLTC (*r* = 0.415, *p* = 0.098) (Figure 6).

### Relationship between EC Metrics and Clinical/Cognitive Performance

**Figure 7:**
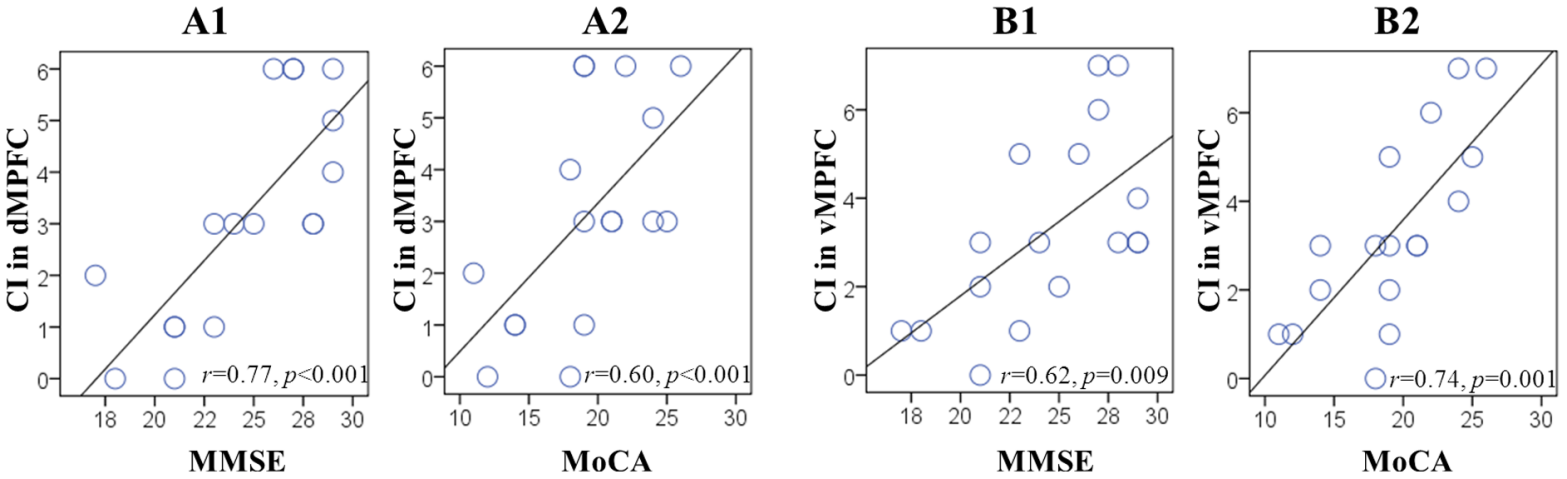
Relationship between CI and individual performance in aMCI. Scatter plots show the significantly positive correlation of CI in the dMPFC with MMSE (*A1*) and MoCA (*A2*) scores, and the significantly positive correlation of the vMPFC with MMSE (*B1*) and MoCA (*B2*) scores. Each circle represents data from one participant.

Finally, we examined the relationship between the convergence degree in regions with between-group EC differences, and individual clinical and cognitive performance. Within aMCI group, we found that CI in the dMPFC and vMPFC significantly correlated (*p*<0.05) with global performance (Figure 7). We also found that CI in the dMPFC and vMPFC was significantly correlated with individual performance on visuospatial executive function and delayed recall tests, and that CI in the rIPL and rLTC correlated with orientation and attention performance, respectively (*p*<0.05). In addition, CI in the vMPFC also showed a trend toward a positive correlation with attention performance (Table 3).

**Table 3:** Spearman correlation between convergence indexes in regions with significantly altered EC and cognitive performance in aMCI group

Region	Spearman correlation coefficient (*p* value)			
	Visuospatial executive function	Attention	Delayed recall	Orientation
dMPFC	0.665 (0.004)^a^	0.337 (0.185)	0.552 (0.002)^a^	0.409 (0.103)
vMPFC	0.700 (0.002)^a^	0.461 (0.063)^b^	0.666 (0.004)^a^	0.299 (0.243)
lIPL	−0.029 (0.913)	−0.032 (0.903)	0.409 (0.851)	0.068 (0.796)
rIPL	−0.022 (0.932)	−0.069 (0.792)	0.024 (0.927)	0.562 (0.019)^a^
rLTC	−0.377 (0.135)	−0.508 (0.037)^a^	−0.079 (0.762)	−0.083 (0.750)

a significant correlation (p<0.05).

b correlation trend (0.05≤p≤0.10).

## Discussion

In this study, we employed Gaussian BN to construct the directed EC patterns within the DMN, and then evaluated changes in patients with diagnosed aMCI. The EC modeling revealed a heterogeneous regional convergence degree across DMN regions in both groups. The between-group comparison found abnormal EC of the DMN in aMCI, including altered directed connectivity weights on several connections that originate from the dMPFC and rLTC, as well as decreased regional CI in the rIPL. Moreover, we found EC changes in aMCI to be correlated with regional FC levels, and that network metrics, including the FC and CI, are associated with patients' clinical and cognitive performance.

### Functional Architecture of the DMN Revealed by BN Analysis

Unlike other traditional fMRI directed connectivity analysis methods, such as structural equation modeling (SEM) [50] and dynamic causal modeling (DCM) [51], the BN learning approach characterizes the dependence structure between multiple interacting regions without requiring any *a priori* assumption of a network model, and provides a global description of a complex system in an exploratory manner [25], [31]. In this study, we employed BN to model and compare the directed EC patterns between DMN regions in the NC and aMCI groups. The BN learning approach revealed similar directed connections and network features in both groups (Figure 2). As differentiated by the CI, the measure defined for the clarification and simplification of local dependence relationships, the 9 identified regions appeared to play different roles in the network. Spontaneous activity in the PCC, dorsal and ventral MPFC, and bilateral IPL, each depends largely on the activity of other DMN regions, while activity in the bilateral LTC and HC is relatively independent of other regions, except for the interdependence between them. It appears that the DMN regions may be organized into two closely interacting subsystems, from the perspective of conditional dependence relationships. This finding agrees with previous heterogeneity studies of DMN regions [52] based on activity [53], functional connectivity [10], [54], and power spectral [52] analysis of the network. These studies demonstrated that the PCC, MPFC, and IPL rank highly in neural activity and oscillatory power, and together constitute a network “core hub” [10], [13], [51], [53], [55]. In our study, activity in these regions was found to depend more on the network, and show a higher degree of local interactivity relative to other DMN regions, including the bilateral LTC and HC. The BN analysis of effective connectivity relationships has therefore deepened our understanding of the functional architecture of the network.

### Altered DMN Connectivity in aMCI

Before comparing the directed connectivity relationships between DMN regions in the NC and aMCI groups, we examined the FC alterations in aMCI subjects. No significant FC difference was detected between the two groups in any of the 9 ROIs. This result appeared to be different from most neuroimaging studies of aMCI which usually showed altered FC of the DMN [6], [20], but was consistent with a recent study of Zamboni et al. [56], in which they also reported unaffected functional connectivity networks including the DMN in aMCI. We speculate that the apparent inconsistency of different studies may be due to the heterogeneous nature of aMCI [57]. However, we found significant correlations between FC variables and clinical/cognitive variables in the dMPFC and rLTC in the aMCI group (Figure 3). Better cognitive performance of aMCI subjects was correlated with higher FC in the dMPFC and lower FC in the rLTC. This result may suggest that although the FC of DMN regions in our aMCI subjects did not show any significant alterations when compared to controls, it may have partly predicted their current cognitive status.

Even though the BN connectivity patterns of the two groups seem similar, the connections between some regions had significant between-group differences in their connectivity weights. We found significantly decreased connectivity from the dMPFC to the bilateral IPL, from the rLTC to the vMPFC, and an increased connection from the rLTC to the rIPL in our aMCI group (Figure 4). An interesting feature of these aberrant connections in aMCI is that they all originated from either the dMPFC or rLTC, in which FC values were shown to be significantly correlated with cognitive variables. In addition, we found that the local CI in rIPL was significantly decreased in aMCI compared with controls (Figure 5), and that it showed a trend toward positive correlation with the FC level in this region (Figure 6). Moreover, we found a significant correlation between the two connectivity attributes in the dMPFC, and an obvious trend in the rLTC in aMCI (Figure 6). This finding indicates that the directed connection alterations and regional interactivity relationship changes evident in DMN regions of the aMCI group have inherent relationships with the FC levels in these regions. In a previous effective connectivity study of the DMN in clinical AD [16], there was also an observation that the aberrant directed connections in AD appeared at the regions with significantly decreased FC, although they paid little attention on the relationship between different connectivity metrics. But the altered connections in their clinical AD appeared to be different from the disrupted connections in our present aMCI subjects. An important potential factor is speculated to be the variation in selection of ROIs between the two investigations. On the premise of identical ROIs definition, a longitudinal study is beneficial for further looking into the relationship between the two connectivity attributes from aMCI to AD. In our study, the results provide new evidence of the disrupted directed connectivity within DMN and suggest an association between directed connectivity changes and regional FC levels in aMCI patients.

The decreased connectivity from the dMPFC to the bilateral IPL revealed in our aMCI subjects demonstrates a typical anterior–posterior disconnection characteristic [58] of the disease. The frontal and parietal regions have been identified as the sites most markedly affected by the neuropathology of AD [59], and the disconnection between them has been suggested as a biomarker of early AD [16], [60]. The decreased dMPFC→l/rIPL connectivity demonstrated here confirms that the disconnection between these regions is also present in the aMCI stage, under the resting state condition. Neuroimaging studies of the DMN have linked the MPFC to self-referential processing [61]. In particular, the dMPFC is involved in self-projection when thinking about others [62], [63], and functions together with lateral parietal areas to support the retrieval and evaluation of self-related information [64], [65]. Altered activation of the dMPFC and IPL regions has been shown to be associated with altered reflective, recollective, and evaluative processes in patients with AD in an fMRI study using a self-assessment task [66]. In addition, some task-centered fMRI studies have also reported that hypoactivation or disconnection of these regions is related to impairments in episodic memory retrieval in patients with aMCI [67], [68]. Presumably, the decreased dMPFC→l/rIPL connectivity under the resting state condition, as demonstrated here, could be a reflection or cause of impairment in these corresponding functions, and may serve as an important imaging marker of aMCI.

In addition to fronto-parietal disconnections, altered connections from the rLTC to the vMPFC and rIPL suggest that aMCI patients also have altered temporo-prefrontal and temporo-parietal pathways. The rLTC is a crucial region involved in the retrieval of episodic memory [69]. A deviant pattern of right temporo-prefrontal circuitry has been previously identified as being associated with AD-related memory deficits [70]. Recent DMN studies have also proposed that the vMPFC is involved in self-projection to the personal past [62], and functions with the LTC to respond to retrieval of autobiographical knowledge [63], [71]. Previous structural and functional neuroimaging studies of AD have found that volume atrophy and abnormal activation in the LTC and prefrontal regions are associated with diminished ability to retrieve episodic aspects of autobiographical memory [72], [73]. As episodic memory impairment is the most outstanding, and earliest neuropsychological sign of AD [74], our finding suggests that the decreased resting state rLTC→vMPFC connectivity could also be an important feature of the disease. Increased connectivity from the rLTC to the rIPL was also detected in aMCI group. This increased connectivity indicates that the activity of the rIPL depends largely on the activity of rLTC. A previous FC study of DMN reported increased FC in these regions in aMCI patients [6]. Although we did not find increased FC in either the rLTC or rIPL, we found a higher FC value in the rLTC, which was correlated with lower clinical and cognitive performance scores in aMCI patients (Figure 3*B* *1* and *B2*). This characteristic and the increased rLTC→rIPL connectivity are suggested to reflect a compensatory mechanism, which is active in aMCI [6], [75].

Finally, we found that the convergence degree in regions with altered EC mentioned above was correlated with individual cognitive performance in aMCI group. In addition to correlations between CI in the dorsal/ventral MPFC and global cognitive abilities measured by MMSE/MoCA (Figure 7), we found more correlations between CI in the dorsal/ventral MPFC, rIPL, and rLTC, with multiple cognitive domains (Table 3). However, the CI in these different regions is correlated with different aspects of individual performance. Particularly, the CI in the dorsal and ventral MPFC is related to individual performance on executive control and delayed memory recall tests; the CI in the rLTC was correlated with patients' attentional performance; and the CI in the rIPL was correlated with individual orientation ability. The relationship of MPFC to delayed recall performance further supports the above speculation that MPFC-related disconnections are associated with memory impairments in aMCI. The LTC also plays a critical role in maintaining sustained attention to verbal inputs [76], and altered connectivity of this region has been previously documented in the dorsal attention network of AD patients [8], [77]. With spatial perception and orientation functions being linked to the IPL [78], it is not surprising to observe the involvement of the rIPL in disorientation problems related to aMCI [79]. Thus, our findings confirm a clinically relevant role for the various DMN regions in the pathology of aMCI, and indicate their potential importance in characterizing the progress of the disease.

### Limitations of the Current Study

Several limitations of the present study deserve mention. First, the BN provides a single snapshot of dynamic neural process, and does not take into account temporal relationships between regions. In addition, the acyclic constraint on BN structure determines that the method cannot disclose reciprocal connections [25]. The dynamic BN [80] which can capture temporal interrelationships of brain regions, and model reciprocal connections, would ideally be utilized to reveal more characteristics of the DMN, and to evaluate the changes in these aMCI patients in future works. Second, although the BN works well for detecting relationships between brain regions, it has been suggested that the directionality of identified connections should be interpreted cautiously [81]. Therefore, the present study has limited the focus on network features, by summarizing the connections in the BN framework, and on the examination and discussion of aMCI-induced EC changes. Third, the present study was performed with a relatively small number of participants. It limited us to provide results with sufficient statistical power. Our findings especially the directed connectivity changes were derived in an exploratory manner and were not corrected by multiple comparisons. Larger independent samples would be needed in future works to increase the statistical power of the findings and to improve the reliability of the conclusions. Fourth, as the head motion has been found to have complex challenges on between-group fMRI connectivity comparisons [43], [44], we still cannot rule out the possibility that our findings might remain confounded by the head motions despite we have applied strategies to reduce motion in the present study. Fifth, the present study focused on exploring the aMCI-related directed connectivity changes in the DMN. Multi-mode MRIs investigations of the directed connectivity in the network are required to disclose the structure-function relationship of the network, and then to explain if the cause of directed connectivity deficits in aMCI is functional, structural or both. Finally, as a cross-sectional study, it is not clear to what extent these EC changes are related to the progressive trajectories of the disease. Follow-up longitudinal studies would be required to investigate the relationship of these EC changes to the progression to AD.

## Conclusions

In summary, using a combined Group ICA and Gaussian BN analysis of resting state fMRI data, we have constructed an effective connectivity network of DMN regions, revealed the organization pattern within the network, and demonstrated abnormal EC of DMN in aMCI patients. Thus, our study provides novel sights into the functional architecture of the DMN, and our findings add to a growing body of work showing the importance of DMN as a neural substrate of aMCI.
